# Histological and Functional Changes Induced by Compression and Decompression Develop Slower in Diabetic Nerves Than in Nondiabetic Nerves

**DOI:** 10.1155/bmri/3777921

**Published:** 2026-09-10

**Authors:** Ignacio Garcia Fleury, Jessica E. Goetz, Douglas C. Fredericks, Emily B. Petersen, Natalie A. Glass, Joseph A. Buckwalter

**Affiliations:** ^1^ Department of Orthopedics and Rehabilitation, University of Iowa, North Liberty, Iowa, USA, uiowa.edu; ^2^ Department Biomedical Engineering, University of Iowa, North Liberty, Iowa, USA, uiowa.edu

## Abstract

**Background:**

Treatment of compressive neuropathies in diabetic patients remains controversial, with variable functional outcomes reported. This study is aimed at establishing a rat model of diabetic nerve compression that is suitable for investigations of diabetic nerve recovery after surgical decompression.

**Methods:**

Twenty male Zucker diabetic fatty obese (diabetic) and 20 lean (nondiabetic) rats underwent surgery to compress the sciatic nerve using a polymer sleeve or sham surgery. Six weeks later, decompression surgery or second sham surgery was performed. Nerve conduction velocities (NCV) and nociception via von Frey filaments (vF) were monitored weekly. Histological measures of nerve structure were compared between groups after 6 weeks of compression and 6 weeks after surgical decompression.

**Results:**

Both diabetic and nondiabetic compressed nerves had significant reductions in NCV immediately postoperatively (*p* < 0.001) and progressive reductions in vF threshold during the compression period (*p* ≤ 0.002). After decompression, vF threshold and NCV increased; however, nerve function did not return to baseline or sham control values in either compressed group. Six weeks of compression significantly reduced axon size (*p* < 0.001) and myelin thickness (*p* = 0.014) in nondiabetic nerves but minimally changed the histological appearance of diabetic nerves. Decompression of nondiabetic nerves increased axon numbers (*p* = 0.005) but did not affect myelin thickness. In diabetic nerves, decompression also increased axon numbers, whereas myelin thickness decreased (*p* = 0.022) to levels similar to nondiabetic nerves.

**Conclusions:**

The histological response of nondiabetic nerves to compression and decompression was more pronounced than the functional response. Diabetic nerves showed a similar reaction to nondiabetic nerves but at a slower rate.

## 1. Introduction

Peripheral compressive neuropathy (PCN) is a condition caused by entrapment of peripheral nerves by various anatomical structures (bones, tendons, and ligaments). This entrapment increases mechanical pressure on the nerve and can impede blood flow, thereby causing local demyelination, axonal degeneration, and loss of sensory and/or motor function [1]. Diagnosis of PCN in diabetic individuals can be particularly challenging, as it may present similarly to, or occur simultaneously with, diabetic peripheral neuropathy (DPN). In contrast to PCN, DPN results from a progressive loss of nerve fibers due to hyperglycemia‐induced metabolic alterations, oxidative stress, and inflammation; although it, too, leads to demyelination, axonal degeneration, and sensory, motor and autonomic dysfunction [2]. However, emerging evidence suggests that diabetic neuropathy may include a secondary compressive component driven by metabolic swelling of peripheral nerves. Activation of the polyol pathway leads to sorbitol and fructose accumulation, increasing intra‐axonal osmotic pressure and nerve enlargement, which predisposes nerves to entrapment within fibro‐osseous tunnels. Therefore, diabetic and nondiabetic compressive neuropathies may share a common mechanical endpoint, with diabetes introducing a metabolic factor that increases susceptibility to compression rather than representing a wholly distinct process. PCN incidence in the diabetic population is as high as 30%, which is significantly greater than the 2%–3% incidence in the general population. DPN presents at a remarkably similar incidence of 35%–45% in the general diabetic population (though up to 66% among insulin‐dependent diabetics) [3]. This overlap in symptoms makes it challenging to diagnose and then manage PCN in diabetic patients.

Carpal tunnel syndrome (CTS) is one of the most common PCNs, and its management in symptomatic patients ranges from conservative options, like splinting and steroid injections, to surgical release of the entrapment. Diabetes is a strong predictor of failure of conservative management of CTS; however, it is challenging to differentiate symptoms of CTS from DPN, and thereby determine if a diabetic patient is likely to benefit from surgical decompression of a mechanically entrapped median nerve [4]. If a diabetic patient can be correctly identified as having CTS, several studies have reported surgical management provides better results than conservative treatment [5]. Nevertheless, many orthopedic surgeons remain reluctant to operate on diabetic individuals because of a higher risk for wound infection [6]. The challenges with diagnosing CTS independent of DPN and increased chances of complications, combined with a lack of clear understanding of the recovery of diabetic individuals after release surgery, have left many unanswered questions about the ability of a compressed diabetic nerve to functionally recover.

This has led to many studies of nerve compression/decompression and diabetes in animal models. For example, it has been shown that 4 weeks of sciatic nerve compression in streptozotocin‐induced diabetic rats significantly decreases withdrawal threshold and motor NCV [7, 8], and 6 weeks after surgical decompression of sciatic nerves in diabetic rats, Semmes–Weinstein monofilament tests and NCV improved [9]. However, although these studies have demonstrated alterations of nerve function in diabetic rats, they typically do not include a nondiabetic control group for comparison, and related studies that do compare between diabetic and nondiabetic nerve responses to compression typically do not relate nerve function to well‐quantitated histological nerve structure [8, 9]. Therefore, the purpose of this study was to use a rat model of chronic nerve compression to evaluate functional and histological recovery after decompression in diabetic and nondiabetic nerves. We hypothesized that recovery of nociception, nerve conduction, and histological structure of previously compressed diabetic nerves would be less complete than that of nondiabetic nerves after decompressive surgery.

## 2. Methods

### 2.1. Experimental Model and Surgical Procedures

With approval from the University of Iowa Office of the Institutional Animal Care and Use Committee (IACUC #7112072), *n* = 20 Zucker diabetic fatty (ZDF) obese rats, (male; strain 370; diabetic, fa/fa) and *n* = 20 age‐matched, nondiabetic‐control‐rats (male, ZDF strain 380; nondiabetic, fa/+) were obtained from Charles River Laboratories (Wilmington, Massachusetts). Rats were housed in pairs in standard conditions (12/12‐h light/dark cycle, 68°F–79°F, 30%–70% humidity) with enriched paper bedding material and ad libitum access to food (Purina #5008 diet) and water. Blood glucose levels were monitored (OneTouch Ultra 2; LifeScan Inc., Malvern, Pennsylvania) in all rats upon arrival, prior to surgeries, and at time of euthanasia, to ensure blood glucose levels > 200 mg/dL for diabetic (ZDF‐obese) rats and < 200 mg/dL for nondiabetic (ZDF‐lean) rats. Rats were divided into four groups of *n* = 10 each: (1) nondiabetic, noncompressed (ND‐NC); (2) nondiabetic, compressed (ND‐C); (3) diabetic, noncompressed (D‐NC); and (4) diabetic, compressed (D‐C).

Glycemia monitoring was conducted on all animals. Serial measurements were obtained at multiple timepoints spanning from 11 to 35 weeks of age. All ZDF‐obese rats demonstrated persistent hyperglycemia, with mean glucose levels consistently exceeding 300 mg/dL and numerous individual readings surpassing 500 mg/dL; several animals reached or exceeded the upper detection limit of the glucometer (> 600 mg/dL). Hyperglycemia was first documented at 11 weeks of age and remained elevated throughout the observation period. ZDF lean rats remained normoglycemic, with blood glucose values consistently below 200 mg/dL.

**Figure 1:**
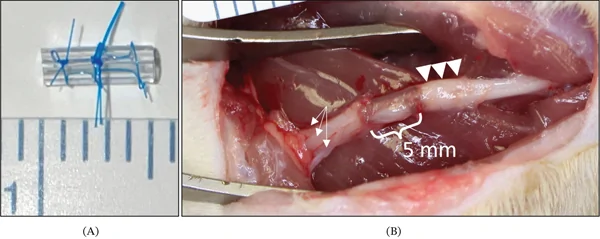
(A) Compression device built with a 5 Fr silicone urinary catheter. Ruler marks indicate millimeters. A longitudinal slit allows for the tube to be placed around the sciatic nerve, and three 8‐0 Prolene sutures (blue) provide evenly distributed closing pressure. (B) Compression device in situ on the left sciatic nerve of a rat after 6 weeks of compression. A bulge in the nerve (arrowheads) is observed proximal to the site of compression, and branching of the sciatic nerve can be appreciated distal to the compression site (arrows).

For surgery, anesthesia was maintained with isoflurane (1%–4%) in oxygen and extended‐release buprenorphine (1 mg/kg; ZooPharm, Laramie, Wyoming) was administered subcutaneously for analgesia. A lateral approach was made through a 2 cm skin incision following a line 0.5–1 cm posterior and parallel to the left femur. Blunt dissection between the fascia of the *biceps femoris* and the *vastus lateralis* muscles exposed the sciatic nerve. A 1.5 cm segment of the sciatic nerve was separated from adjacent structures, and in compression groups (ND‐C and D‐C), a 5‐mm long custom compression device fabricated from a 5 Fr (1.57 mm diameter) pediatric silicone urinary catheter (Coloplast A/S, Humlebaek, Denmark) was placed around the exposed nerve. The nerve was cradled into the open tube, and the tube was pulled closed with three 8‐0 Prolene sutures to apply circumferential pressure to the nerve (Figure 1). In uncompressed groups (ND‐NC and D‐NC), the nerve was manipulated for an equivalent amount of time. The nerve was then returned to its anatomic position, the skin closed with a subcutaneous buried 5‐0 Vicryl, and 0.1 mL of 0.25% bupivacaine was injected locally following skin closure.

Six weeks after surgery, half of the rats from each group were randomly selected and euthanized. The remaining rats (*n* = 5 in each group) underwent a second surgery. In ND‐C and D‐C groups, the nerve was decompressed by releasing the sutures holding the compression device, and the silicone tube was removed. In the ND‐NC and D‐NC groups, a second sham surgery was performed during which the nerve was manipulated gently with microsurgical forceps for an equivalent time as required to remove the compression device. The nerve was replaced in its anatomic position, and the skin was closed. Six weeks later, all remaining animals were euthanized for analysis.

All institutional and national guidelines for the care and use of laboratory animals were followed throughout the study. Veterinary staff monitored the animals twice daily, evaluating physical appearance, behavior, activity, eating/drinking habits, urination/defecation habits, surgical site healing, and limb usage. Altered operative limb usage (limb dragging, abnormal digit/hock position, and reduced weight‐bearing) was noted in some animals after compression surgery; however, the condition did not appear to adversely impact the animal and resolved within a few weeks. Urinary abnormalities (hematuria, balanoposthitis, posthitis, and hematoma formation within urinary bladder lumen) were noted in 13/40 rats, with 10/13 occurrences in diabetic animals. Most cases resolved with fluid therapy/diuresis (7–10 mL/kg, normal saline or lactated Ringer′s solution administered twice daily), antibiotic (enrofloxacin, 5–10 mg/kg, PO, twice daily) and/or topical antifungal (miconazole), and short course of a nonsteroidal anti‐inflammatory medication (meloxicam, 1–2 mg/kg, PO). There was no humane endpoint associated with surgery‐related events (abnormal limb usage); however, in *n* = 2 diabetic animals, a humane endpoint was reached when the urinary bladder palpated turgid, enlarged, and no urine could be expressed during the examination. One additional diabetic animal with urinary abnormalities was found dead in its pen. Data from these animals not reaching the study timepoints (*n* = 3) were excluded from analysis.

### 2.2. Functional Testing In Vivo

**Figure 2:**
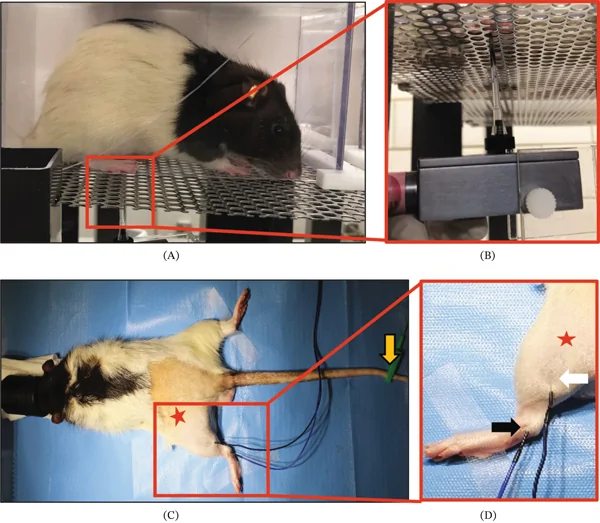
(A) Mechanically induced nociception (von Frey) testing set up. All animals were acclimated to a plexiglass box with wire mesh floor prior to testing. (B) An electronic von Frey aesthesiometer was applied to the paw through the mesh until eliciting a withdrawal response. (C) Nerve conduction velocity measurement set up illustrating the stimulation site proximal to the area of interest (star), ground electrode (yellow arrow down), (D) reference electrode (white left arrow), and recording electrode (black right arrow).

To test nociception, von Frey (vF) testing was performed using an electronic aesthesiometer (IITC Life Sciences, Woodland Hills, California) with a rigid tip to measure response to mechanical stimuli. For testing, rats were placed into individual plexiglass containers with a wire mesh bottom (Figure 2) and allowed to acclimate for 15 min. The aesthesiometer tip was pressed against the plantar surface of the hindfoot until the animal withdrew the paw. Load application was alternated between the left and right hindfoot to acquire four measurements per foot, which were averaged to provide a single left and right foot value for each animal at each evaluation timepoint. Rats were acclimated to testing for 3 weeks before surgery, and baseline values for each animal were established preoperatively. vF testing was then performed three times per week after surgery and averaged into weekly postoperative nociception values.

Nerve conduction velocities (NCVs) in the operative limb were measured noninvasively using a VikingQuest EMG (Natus Neurology Inc, Middleton, Wisconsin). A recording electrode was placed on the heel, a reference electrode on the gastrocnemius, and a ground lead on the tail (Figure 2). A stimulating electrode was used proximal to the compression site to stimulate incremental impulse intensity until a maximal and artifact‐free compound muscle action potential (CMAP) motor response was evoked (range 30–80 mA and duration of 0.1 ms; amplitude range 10–30 mV) [10]. NCVs were collected under isoflurane inhalation anesthesia immediately pre‐ and postoperatively; at Weeks 1, 2, and 4 postoperatively; and either immediately prior to euthanasia or immediately before and after the secondary surgery at 6 weeks. For animals with a second surgery (decompression or sham), NCVs continued to be collected at Weeks 7, 8, and 10, and prior to euthanasia at Week 12. Testing at all timepoints was performed by the same individual to standardize electrode placement.

### 2.3. Euthanasia and Tissue Harvest

While under isoflurane anesthesia, rats were euthanized via an intracardiac injection of euthanasia solution containing pentobarbital, with bilateral thoracotomy to confirm euthanasia. The sciatic nerve was immediately fixed in situ for a period of 30 min using Karnovsky′s fixative changed every 10 min. Compressed nerves were excised with cuts 15–20 mm proximal to and distal to the compression site, and an equivalent length/location of nerves that underwent sham surgery and the uninjured contralateral limb were harvested. Nerves were pinned straight on a piece of cork while maintaining proximal/distal orientation, and completely fixed in Karnovsky′s fixative [11].

### 2.4. Histological Analysis

Nerve specimens were embedded in resin to preserve tissue organization and myelin structure [11]. Six‐micron thick sections through the compressed (or spatially equivalent location on uncompressed nerves) were stained with toluidine blue [11] and digitized through a 40x objective (161 nm/pix) of a stage scanner microscope (VS110; Olympus America Inc., Center Valley, Pennsylvania). To objectively quantify compression‐related differences in the microscopic appearance of the nerves, a custom, automated analysis algorithm was developed in MATLAB (The MathWorks; Natick, Massachusetts). First, a location within the nerve free from blood vessels and other nonaxonal structures was manually selected. A 600x600‐pixel (approximately 100x100‐micron) tissue region of interest (ROI) centered on that location was automatically extracted for analysis. Utilizing a combination of thresholding and morphology‐based functions, the darker‐staining myelin sheath was identified and used to separate the ROI into myelin area, interior axonal area, and total axonal area (myelin area plus interior axonal area). The percent myelinated area and percent axon area were calculated relative to the tissue ROI area, and the number of axons was automatically totaled within the ROI. To calculate average myelin thickness, a series of search rays perpendicular to the local interior axon perimeter were projected through a given axon′s surrounding myelin layer and averaged into a per axon value. Values for axons within −1 to +2 standard deviations of the mean axon area in the given nerve were averaged to calculate the nerve′s average myelin area.

### 2.5. Statistical Analysis

Histological measures of axon number, percent myelinated area, percent axon area, and average myelin thickness and functional measures of NCV and vF threshold were compared between experimental and contralateral control limbs and between study groups. To evaluate the relationship between compression/noncompression and time for NCV and vF measurements, a mixed model was used (SAS 9.4; Cary, North Carolina) with the significance level set as *p* < 0.05 [12]. Two‐way ANOVA with Tukey′s multiple pairwise comparison tests and a multiplicity adjusted *p* value < 0.05 was used to detect differences in histological outcomes between groups (Prism v10.3.0, GraphPad Software, LLC; Boston, Massachusetts).

## 3. Results

### 3.1. Mechanical Stimulus‐Induced Nociception: vF Testing

**Figure 3:**
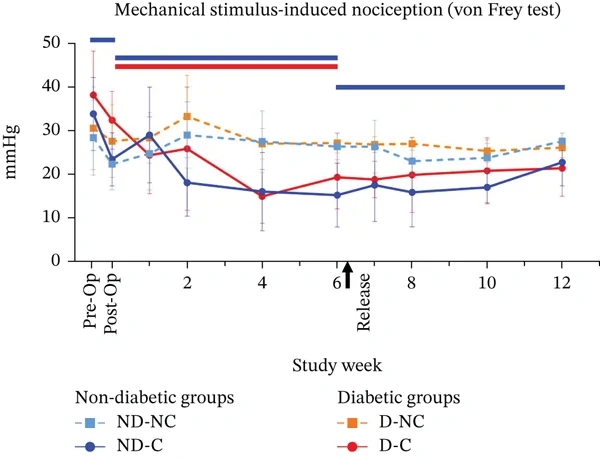
Longitudinal plots of mechanically induced nociception (von Frey) test measurements for all experimental limbs. Symbols indicate group mean, and vertical error bars are standard deviations. In the compressed groups (solid lines), decompression surgery was performed 6 weeks after index surgery to place the compression device. In the sham surgery group (dashed lines), nerve mobilization was performed at index surgery and again 6 weeks later. Release was performed at 6 weeks after acquisition of the 6‐week data. Horizontal bars indicate statistically significant (*p* < 0.05) differences between the timepoints marked by the ends of the lines.

Index surgery (compression or sham) reduced the vF stimulus threshold in all groups (Figure 3), but the reduction was only statistically significant in the nondiabetic compressed group (ND‐C *p* = 0.006). Compression did not have an immediately significant effect on vF threshold in either diabetic (D‐NC vs. D‐C *p* = 0.602) or nondiabetic (ND‐NC vs. ND‐C *p* = 0.388) animals; however, after 6 weeks of compression, the vF threshold was significantly lower than immediate postoperative values in both ND‐C (*p* = 0.002) and D‐C (*p* < 0.001) nerves. vF thresholds were minimally affected by second surgery (sham or decompression). By 6 weeks after decompression, the ND‐C thresholds had significantly increased relative to the compressed values (*p* < 0.001), whereas no other group had significantly different vF thresholds (Figure 3).

### 3.2. NCV

**Figure 4:**
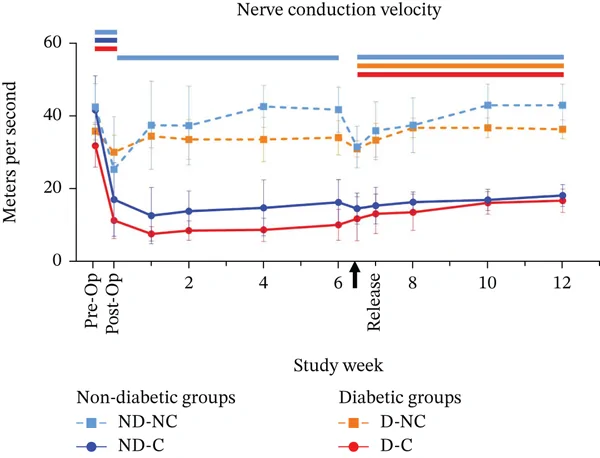
Longitudinal plots of nerve conduction velocity for all experimental limbs. Symbols indicate group mean, and vertical error bars are standard deviations. Horizontal bars indicate statistically significant (*p* < 0.05) differences between the timepoints marked by the ends of the lines. Data acquired at the “release” timepoint were also obtained at 6 weeks, after second surgery but while the animal was still sedated for the second surgery (release or sham).

All groups had significantly slower NCV (Figure 4) immediately after surgery except for D‐NC (*p* = 0.060). Compression significantly slowed NCV in diabetic nerves (D‐NC vs. D‐C *p* < 0.001, immediately post‐op), even though preoperative NCV was already slower than in nondiabetic nerves. Six weeks after initial surgery, both NC groups recovered to preoperative values. In contrast, the initial reduction in NCV for both compressed groups was sustained, and NCV values after 6 weeks of compression were not significantly different than immediately postoperatively (ND‐C *p* = 0.819; D‐C *p* = 0.737). Second surgery again reduced NCV in all groups except D‐C (Figure 4), and NCV similarly increased after second surgery in the D‐NC (*p* = 0.011) and ND‐NC (*p* < 0.001) to reach preoperative values. Following decompression, D‐C nerves demonstrated a significantly increased NCV (*p* = 0.040), whereas increases in NCV after decompression in the ND‐C group did not reach statistical significance (*p* = 0.131).

### 3.3. Nerve Histological Appearance

**Figure 5:**
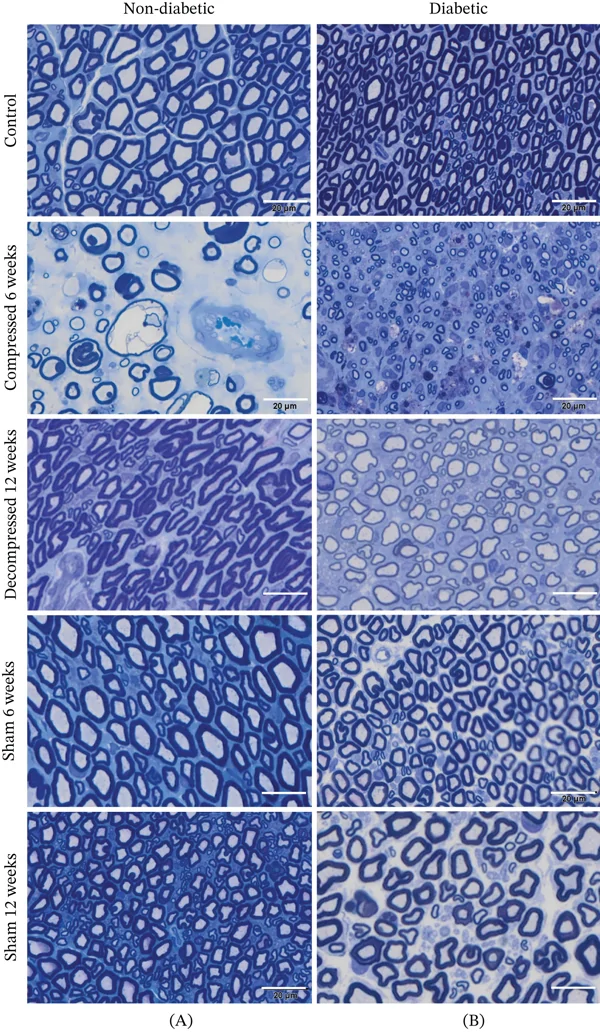
Illustrative toluidine blue‐stained nerve from each experimental group. White scale bars are 20 *μ*m. (A) Uninjured, contralateral nerves from nondiabetic rats (normal nerves) demonstrated tightly packed, polygonal‐shaped axons with uniform myelin thickness that corresponded to axon cross‐sectional area. There was minimal interstitial space, minimally cellular collapsed blood vessels, and less discernable perineurial divisions with maintained epineural thickness. After 6 weeks of compression, myelin thickness was markedly reduced, with clear signs of delamination. Interstitial space was abundant, perineurial barriers were absent, and axons adopted a more circular appearance with highly variable diameters. Blood vessels appeared turgid with trophic changes in the wall and high cellular content in the lumen. Epineurium appeared thickened with vascular infiltration. By 12 weeks (6 weeks after decompression), most parameters had improved toward normal, although some trophic changes in blood vessels and thickening of the epineurium and perineurium remained. Sham surgery had minimal effects on nerve appearance at either the 6‐ or 12‐week timepoint. (B) Normal nerves from the contralateral limbs of diabetic rats demonstrated smaller diameter axons, thinner myelin, and a less tightly packed organization. After 6 weeks of compression, all markers of injury described for the nondiabetic nerves were evident, but to a lesser degree: Axons maintained thick myelin sheaths while adopting a circular shape, perineurial divisions remained discernable, but epineurium thickness was enlarged. However, all injury markers were still present by 12 weeks (6 weeks after decompression). Interstitial space decreased slightly, axons remained rounded, myelin thickness remained thin, and blood vessels maintained trophic changes in their walls with a high cellular content in their lumen. Sham surgery had little effect at 6 weeks, and multiple sham surgeries increased heterogeneity in the nerve appearance at 12 weeks.

**Figure 6:**
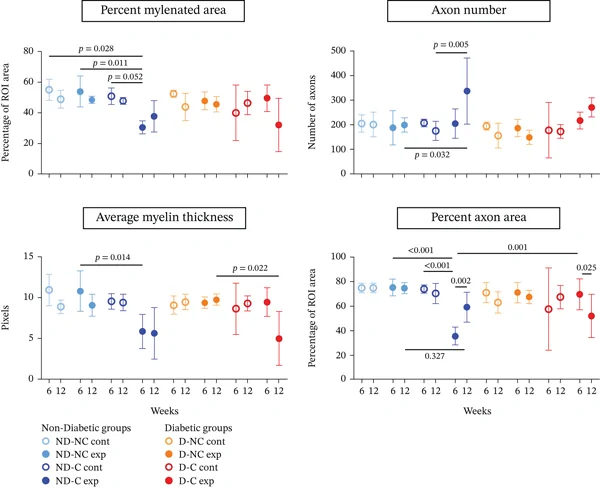
Quantitative histology data from contralateral (open circles) and experimental (solid circles) limb nerves. Nondiabetic nerves appeared to respond faster to compression by increasing axon number and losing myelin thickness, resulting in reduced percent myelinated area and reduced percent axon area after 6 weeks of compression. Six weeks after decompression (12‐week timepoint), nondiabetic nerves had an increased number axons, resulting in an increased percent myelinated area and percent axon area, despite myelin thickness remaining low. Diabetic nerves appeared to respond similarly, but slower, with decreases in myelin thickness, myelinated area, and axon area not developing until 12 weeks.

Sham surgery had minimal effect on the appearance of diabetic or nondiabetic nerves at either the 6‐ or 12‐week timepoint (Figure 5). Changes in histological appearance due to compression and decompression developed and resolved faster in the nondiabetic nerves than in diabetic nerves. The percent myelinated area in the ND‐C nerves decreased significantly (Figure 6) after 6 weeks of compression compared with both the associated contralateral (*p* = 0.052) and to the sham experimental (ND‐NC: *p* = 0.011) nerves. Six weeks after decompression, the percent myelinated area had not significantly increased versus that of 6 weeks of compression (*p* = 0.995). In diabetic nerves, there were no significant differences in percent myelinated area associated with sham surgery or compression at 6 weeks, but there was a slight decrease in percent myelinated area in decompressed nerves at 12 weeks (*p* = 0.247; vs. 6 weeks). Changes in average myelin thickness paralleled those of percent myelinated area. Thickness significantly decreased in ND‐C nerves at 6 weeks relative to sham surgery values (ND‐NC; *p* = 0.014); however, D‐C nerve myelin thickness remained similar to control values at 6 weeks. By 12 weeks, D‐C nerve myelin had become significantly less thick than D‐NC values (*p* = 0.022), reaching comparable thickness with decompressed ND‐C nerves in which the myelin thickness remained decreased.

The number of axons remained consistent across diabetic and nondiabetic contralateral nerves and nerves with sham surgery at all timepoints. There were significantly more axons present in decompressed ND‐C nerves compared with the associated contralateral (*p* = 0.005) and to ND‐NC nerves (*p* = 0.032) at the same 12‐week timepoint. A similar trend was present for the diabetic nerves, but the increased number of axons after decompression did not reach significance (*p* = 0.994 vs. D‐C at 12 weeks). The percent axon area is the combination of axonal and myelinated areas in the ROI. In ND‐C nerves, percent axon area was significantly lower than in the associated contralateral (*p* < 0.001) and ND‐NC group (*p* < 0.001) at the same 6‐week timepoint. After decompression, ND‐C percent axon area increased to levels not significantly different than sham surgery groups (ND‐NC; *p* = 0.327) at the same 12‐week timepoint. Diabetic nerves demonstrated the opposite trend, with no significant difference in percent axon area in compressed nerves, but a significant loss in decompressed nerves (*p* = 0.025) (Figure 6).

## 4. Discussion

Appropriate treatment of PCN in diabetic individuals is confounded by questions about impaired healing and the ability of nerves damaged by diabetes to recover after surgical decompression. This work utilized a rat model of peripheral nerve compression that was distinctive in its relevance to PCN conditions (vs. models of nerve transection or crush injury [3, 8, 9, 13–19],) to determine the potential for recovery in diabetic nerves as compared with nondiabetic nerves. In this PCN model, surgical insult alone was found to induce transient decreases in mechanical nociception (vF) thresholds and NCV that recovered to preoperative values in both diabetic and nondiabetic rats soon after surgery. Compressed nerve function was reduced to similar levels in both diabetic and nondiabetic rats, and functional recovery after decompression was both modest and similar across diabetes states. Compression‐ and decompression‐induced changes in histological appearance were amplified in the nondiabetic nerves. Diabetic nerves appeared to be following the same trajectory of thinning myelin and increasing number of axons but on a slower timeline than nondiabetic nerves. Increases in nerve function after decompression appeared more related to an increasing number of axons, given the myelin thickness in both diabetic and nondiabetic nerves remained substantially thinner 6 weeks after decompression. The delayed structural response observed in diabetic nerves may reflect impaired adaptation to compression rather than a fundamentally different injury pattern. Metabolically induced nerve swelling and microvascular dysfunction have been proposed to increase susceptibility to compression and impair recovery.

NCV and vF thresholds in uncompressed sham operated nerves, both ND‐NC and D‐NC, recovered quickly to baseline function from reductions associated with surgical insult. In contrast, compressed nerves (ND‐C and D‐C) demonstrated further functional decline while the nerve remained compressed. This demonstrated that the model accurately reflects the clinical condition of progressive loss of sensory and motor function with continuous nerve compression characteristic of PNC [20]. Nerves that were decompressed at 6 weeks (ND‐C and D‐C) did not return to baseline function by 12 weeks (6 weeks after decompression), although both groups demonstrated weekly improvement in both NCV and vF threshold after decompression. It is unclear whether nerve function in either group would recover to baseline if followed for a longer time after decompression. The improvement in diabetic nerve function after decompression is somewhat inconsistent with previous reports of incomplete recovery in motor NCV in diabetic rats after decompression but similar to the reported partial recovery of nociception (Hu et al. [9]). Our results are most similar to those of Wang et al. ([15]), who showed diabetic and nondiabetic rats alike recovered both sensory and motor nerve function postdecompression, although diabetic rats only showed partial improvement. Finally, Zemp et al. ([8]) described that diabetic nerves displayed progressive loss in function and structure over time, indicating that diabetic nerves have a limited capacity for functional recovery—even with decompression. Overall, the comparative analysis reveals a consistent theme of impaired functional recovery in diabetic nerves across the current literature.

Histological analysis showed that nondiabetic nerves exhibited more pronounced structural changes compared with diabetic nerves. Compression significantly decreased, and then decompression significantly increased axonal area in nondiabetic nerves. Diabetic nerve response to compression was slower than that of normal nerves. The higher number of axons in both the ND‐C and D‐C nerves after decompression could be an indication of an axonal sprouting [21], a critical component for recovery and regeneration. In ND‐C nerves, myelin thickness significantly decreased, and remained decreased even after decompression. Myelin in D‐C nerves did not change because of compression but began to thin to the same degree as the ND‐C between 6 and 12 weeks. This response is like that of Hu et al. [9] who found that diabetic nerves showed persistent myelin degeneration and axonal swelling postcompression, with minimal improvement after decompression. Siemionow et al. [16] emphasized that early decompression preserved nerve structure in diabetic models, suggesting that timing is critical for successful recovery, a variable not addressed in our research. Tuck et al. [19] showed that reduced endoneurial blood flow and oxygen tension contribute to persistent structural abnormalities in diabetic nerves, providing a mechanistic possible explanation for our observed findings of slower response to compression. Zemp et al. [8] and Soo‐Hwan et al. showed decreased myelin thickness and mitochondrial damage, which remained unchanged despite therapeutic interventions, supporting the maintained histological deficits seen in the current research in diabetic animals. Surgical decompression reduces intraneural pressure and restores local mechanical conditions, as demonstrated in pressure‐based studies of fibro‐osseous tunnels, which may facilitate partial functional recovery even in the absence of complete histological normalization. Overall, although our study corroborates the limited recovery of myelin thickness after decompression of a previously compressed nerve and axonal sprouting after compression, it also emphasizes commonalities and potential time‐delayed response of decompressed diabetic nerves relative to nondiabetic nerves. Notably, functional improvements after decompression occurred despite persistent abnormalities in myelin thickness, suggesting that functional recovery may be mediated by axonal remodeling rather than full structural restoration.

This study has several limitations. The use of only five animals per group at each timepoint limited the statistical power; however, significant differences were still observed, providing valuable pilot data for future studies. Diabetic rats likely had larger nerves, experiencing slightly greater compression from the standardized device, but they showed similar functional and histological outcomes as nondiabetic nerves, indicating comparable compression effects. The consistency of vF values at later timepoints suggests behavioral adaptation, highlighting vF testing′s insensitivity for longitudinal nerve function assessment. Finally, the 6‐week postdecompression follow‐up limits conclusions about long‐term nerve recovery.

## 5. Conclusions

Peripheral nerve compression represents a shared mechanical pathway in both diabetic and nondiabetic conditions. In diabetes, metabolic alterations may increase susceptibility to compression and delay structural adaptation. Although decompression improves nerve function, persistent histological abnormalities suggest incomplete biological recovery. These findings support the importance of timing and indicate that functional improvement may not reflect full structural normalization. The study underscores the decreased responsiveness of diabetic nerves to morphological changes associated with compression/decompression and suggests a prolonged timeline to achieve improved microstructure after decompression. However, functional deficits and recovery were similar between diabetic and nondiabetic animals, suggesting future investigations could contribute to pinpointing a “point of no return” and assessing the efficacy of early surgical decompression in diabetic patients. Future investigations can focus on finding the specific timeline for histological recovery in both diabetic and nondiabetic nerves following decompression, with an emphasis on identifying when structural features such as myelin thickness and axonal area return to baseline levels. This will help establish the relationship between structural normalization and the recovery of nerve function before compression.

## Funding

This study was funded by the University of Iowa Department of Orthopedics and Rehabilitation.

## Ethics Statement

All animal procedures in this study were conducted in accordance with institutional and national guidelines for the care and use of laboratory animals. Ethical approval for the study was obtained from the University of Iowa Office of the Institutional Animal Care and Use Committee (IACUC) (Approval Number: 7112072). All procedures followed ethical regulations, and veterinary staff monitored the animals twice daily to ensure their welfare.

## Consent

This study did not involve human participants; therefore, informed consent was not required.

## Conflicts of Interest

The authors declare no conflicts of interest.

## Data Availability

The data that support the findings of this study are available from the corresponding author upon reasonable request.
